# Targeting Tunable Physical Properties of Materials for Chronic Wound Care

**DOI:** 10.3389/fbioe.2020.00584

**Published:** 2020-06-11

**Authors:** Yuzhen Wang, Ubaldo Armato, Jun Wu

**Affiliations:** ^1^Research Center for Tissue Repair and Regeneration Affiliated to the Medical Innovation Research Department and 4th Medical Center, PLA General Hospital and PLA Medical College, Beijing, China; ^2^PLA Key Laboratory of Tissue Repair and Regenerative Medicine and Beijing Key Research Laboratory of Skin Injury, Repair and Regeneration, Beijing, China; ^3^Research Unit of Trauma Care, Tissue Repair and Regeneration, Chinese Academy of Medical Sciences, 2019RU051, Beijing, China; ^4^Department of Burn and Plastic Surgery, Air Force Hospital of PLA Central Theater Command, Datong, China; ^5^Histology and Embryology Section, Department of Surgery, Dentistry, Pediatrics and Gynecology, University of Verona Medical School Verona, Verona, Italy; ^6^Department of Burn and Plastic Surgery, Second People's Hospital of Shenzhen, Shenzhen University, Shenzhen, China

**Keywords:** chronic wounds, stem cells, mechanical properties, structural properties, nanotechnology

## Abstract

Chronic wounds caused by infections, diabetes, and radiation exposures are becoming a worldwide growing medical burden. Recent progress highlighted the physical signals determining stem cell fates and bacterial resistance, which holds potential to achieve a better wound regeneration *in situ*. Nanoparticles (NPs) would benefit chronic wound healing. However, the cytotoxicity of the silver NPs (AgNPs) has aroused many concerns. This review targets the tunable physical properties (i.e., mechanical-, structural-, and size-related properties) of either dermal matrixes or wound dressings for chronic wound care. Firstly, we discuss the recent discoveries about the mechanical- and structural-related regulation of stem cells. Specially, we point out the currently undocumented influence of tunable mechanical and structural properties on either the fate of each cell type or the whole wound healing process. Secondly, we highlight novel dermal matrixes based on either natural tropoelastin or synthetic elastin-like recombinamers (ELRs) for providing elastic recoil and resilience to the wounded dermis. Thirdly, we discuss the application of wound dressings in terms of size-related properties (i.e., metal NPs, lipid NPs, polymeric NPs). Moreover, we highlight the cytotoxicity of AgNPs and propose the size-, dose-, and time-dependent solutions for reducing their cytotoxicity in wound care. This review will hopefully inspire the advanced design strategies of either dermal matrixes or wound dressings and their potential therapeutic benefits for chronic wounds.

## Introduction

Infections, diabetes, or radiation exposures promote chronic wounds. About 1–2% of the population in developed countries suffer from chronic wounds throughout their lifetime (Dovi et al., 2003; Satish et al., 2017). In China, the prevalence rate of chronic wounds was 1.7‰ among hospitalized patients based on the latest cross-sectional epidemiological survey (Jiang et al., 2011). The feet of diabetic patients usually suffer more as compared with other bodily parts because of further aggravations due to persistent bacterial infections, to sympathetic nerve dysfunctions, and to the continuous frictions of walking (Akash et al., 2020). Approximately one diabetic patient in six suffers from chronic foot wounds (Bakker et al., 2016). Among them, each year almost one million diabetic patients have to undergo lower limb amputation (Boulton et al., 2005). With the aging of the global population, chronic wounds are becoming a worldwide growing medical burden.

Traditional therapeutic approaches to chronic wounds are unsatisfactory because patients suffer prolonged pain as well as unavoidable upshots such as scarring and physical dysfunctions. Commonly used therapeutic approaches are chemical strategies, such as drugs, growth factors, agonists, or inhibitors of critical signaling pathways. However, recent advances in the fields of stem cells and nanotechnology highlighted some knowledge-updating discoveries of physical properties (i.e., mechanical-, structural-, and size-related properties) in directing endogenous stem cell fates (Jiang et al., 2018) and fighting bacterial resistance (Bhattacharya et al., 2019), which might play major roles in chronic wound healing.

The World Health Organization reported that about 265,000 deaths occur every year caused by burns or lack of appropriate treatments including skin substitutes or wound dressings (Das et al., 2016; Khorasani et al., 2018). Generally, skin substitutes aim at replacing missing tissues with gradually degrading dermal matrixes (Ramanathan et al., 2017), while wound dressings cover wound beds acting as temporary mechanical barriers to prevent bacterial infections and to avoid the loss of water and nutrients (Kalantari et al., 2020). Herein, we respectively discuss how the mechanical and structural properties of dermal matrixes affect stem cell behavior, as well as the application of nanoparticle-based (size-related properties) wound dressings for treating infections of chronic wounds. Moreover, we also discuss some worries about mechanical- and structure-related regulation of stem cells. We also address current biosafety concerns about nanoparticles (NPs) and examine workable solutions to reduce their cytotoxicity.

## Chronic Wound Healing

### Endogenous Stem Cells

**Figure 1:**
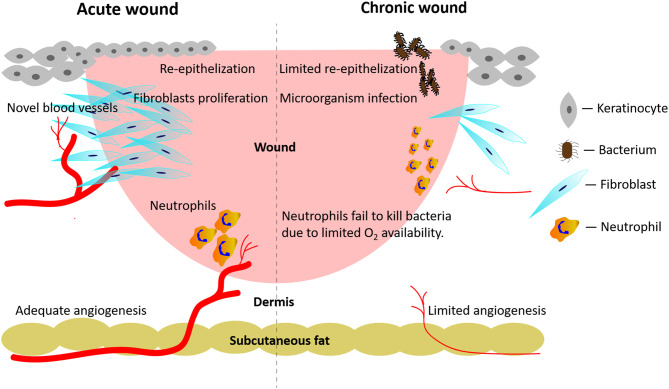
Pathological mechanisms active in acute wounds and chronic wounds, respectively. Acute wounds **(Left Side)**: an adequate angiogenesis promotes re-epithelialization, fibroblasts' proliferation, and neutrophils' anti-infection activities. Chronic wounds **(Right Side)**: persistent local bacterial infections hinder the formation of novel blood vessels. In turn, the restricted angiogenesis hampers fibroblasts' proliferation and the neutrophils' anti-infection activities.

Concerning acute wounds, healing processes are singled out in stages such as hemostasis, inflammation, novel tissue generation, and remodeling, which successively appear and overlap one after the other. Endogenous stem cells (i.e., endothelial progenitor cells, epidermal stem cells, etc.) can both undergo self-renewal and differentiate into one or multiple lineages to repair tissue losses (Kanji and Das, 2017). However, infection, diabetes or radiation exposure may disrupt the well-orchestrated stem cell behaviors and result in chronic wounds (Figure 1). Even worse, continuing pathological risks (i.e., bacterial infection, high blood glucose levels, local continuous pressure or friction, etc.) hinder the healing of wounds, which become chronic when they did not show any sign of improved healing after 30 days (Kim et al., 2018).

Endogenous endothelial progenitor cells, which are either resident in wound environments or originate from bone marrow, can promote wound healing via angiogenesis (Kanji and Das, 2017). Angiogenesis involves the sequential occurrence and overlap of various stages, i.e. the activation of endothelial cells, the degradation of the endothelial cells' basement membranes, and the sprouting and ripening of newly-formed vascular structures (Huang et al., 2019). Various alterations of oxygen, nutrients, metabolites' levels, and inflammatory events easily impact on the whole angiogenic process. For example, diabetic wounds easily suffer from hypoxia (or reduced oxygen supply) due to inadequate angiogenesis and a concurring vascular dysfunction and neuropathy. Even more worrying, diabetic wounds usually exhibit higher oxygen consumption rates causing a further lessening of available oxygen *in situ* (Hopf and Rollins, 2007). Concurrently, an inadequate angiogenesis due to an impaired function of endogenous endothelial progenitor cells restricts the proliferation of fibroblasts and their collagen deposition by affecting the hydroxylation of proline and lysine residues, which impacts on scarring's outcomes (Figure 1; Desmet et al., 2018).

Endogenous epidermal stem cells with different lineages inhabit the basal layers, interfollicular epidermis, sebaceous glands, eccrine sweat ducts, or hair follicles bulges. During wound healing processes, epidermal stem cells are capable of differentiating into multiple lineages and of repopulating other epidermal components (Haensel et al., 2020). In chronic wounds, most epidermal stem cells show a blunted self-renewal or have been destroyed together with the missing deep tissue. In this case, re-epithelialization typically occurs from the peripheral edges of chronic wounds, being mediated by the recruitment of mobilized stem cells from wound-adjacent stem cell sources (Vagnozzi et al., 2015). Therefore, regulating endogenous stem cells behaviors plays a vital role in chronic wound care.

### Infection

Persistent infections help bring about chronic wounds, which are all susceptible to a contaminating localization of microorganisms. The proliferation coupled with toxins release of the localized microorganisms causes inflammatory reactions in the host. Microorganisms can form polymicrobic biofilms, which are one of the mechanisms underlying antibiotic resistance. If left untreated, the infected wounds do not heal (Figure 1).

The infecting organisms, most often bacteria, can easily attach to the wound bed and enter into the blood system because the skin barrier is no longer present. *Staphylococcus*, a genus of Gram-positive bacteria which includes more than 40 species, is the most common type of infectious agent found in burn wounds (Dhanalakshmi et al., 2016). The methicillin-resistant *Staphylococcus aureus* (MRSA) is the most common antibiotic-resistant microorganism responsible for hospital-acquired infections (Dantes et al., 2013). Other bacteria genuses found in wounds environment are *Pseudomonas aeruginosa, Klebsiella pneumoniae, Proteus mirabilis, Streptococcus faecalis*, and others more. Besides, an alteration of intestinal flora might cause the gut bacteria to trespass into circulating blood; this has become a current research hotspot and has attracted a lot of attention (He et al., 2019).

In chronic wounds, inadequate angiogenesis due to functionally impaired endogenous endothelial progenitor cells might further reduce the innate anti-infection capabilities. When wounds have become infected, the phagocytosis of the involved pathogens by leukocytes will trigger the respiratory burst process resulting in the release of massive amounts of bactericidal reactive oxygen species (ROS). Reportedly, the respiratory burst process consumes about 98% of the oxygen in neutrophils (Bryan et al., 2012). A lack of oxygen in chronic wounds impairs the anti-infection abilities of neutrophils (Figure 1).

Usually, diabetic patients show high blood glucose levels due to reduced autologous insulin secretion or increased insulin resistance, which result in multiple metabolic dysfunctions. Several underlying pathological factors also contribute to the delayed healing of diabetic wounds, such as local persistent bacterial infections coupled with excessive levels of pro-inflammatory cytokines, proteases, ROS (Frykberg and Banks, 2015), and with a worsening vascular dysfunction combined with the cells' inability to respond to pro-reparative stimuli (Kim et al., 2018).

## Mechanical Properties

Mechanical properties of dermal matrixes include stiffness, elastic modulus, tensile strength, viscoelasticity, stress stiffening effects, stress-relaxation rate, and more. Recent studies about mechanical-related regulation of stem cells and elastin-based dermal matrixes highlighted the practical solutions for chronic wound care.

### Mechanical-Related Regulation During Wound Healing

Clinical practice in wound healing has shown that higher tension sutures of surgical wounds increase scar tissue formation, and that the stress and stiffness of wound fixation could also affect wound healing speed and quality. However, the underlying mechanism is still unclear.

Recently, mechanical signals have increasingly shown an overarching ability to regulate stem cell characteristics and lineages. Among mechanical signals, stiffness-related control of cell fates has been studied extensively. For example, mesenchymal stem cells (MSCs) show distinctive differentiation patterns when cultured in matrixes of tunable stiffness. MSCs changed their lineage specification into neurons, myoblasts or osteoblasts when cultured in polyacrylamide hydrogels with a tunable stiffness gradient varying from 0.1 to 25 kPa (Engler et al., 2006). In other words, MSCs “felt” matrixes stiffness and then “chose” their direction of differentiation.

Further research showed that the transcriptional co-activator with PDZ-binding motif (TAZ) and Yes-associated protein (YAP) played vital roles in the stiffness-related regulation of stem cell fates (Yang et al., 2014). YAP/TAZ are two highly related downstream readers and transcriptional regulators of mechanotransduction, serving as molecular “beacons” of cellular responses to surrounding mechanical stimuli (Brusatin et al., 2018). Reportedly, the AT-rich interactive domain-containing protein 1A (ARID1A) is located in the chromosome 1p36 region acting as a tumor suppressor gene (Guan et al., 2011). The ARID1A belongs to the switch/sucrose non-fermenting (SWI/SNF) chromatin remodeling complex, which encodes a large nuclear protein involved in chromatin remodeling (Guan et al., 2011). The full activation of YAP/TAZ activity needs to meet two requirements: i.e., both the promotion of YAP/TAZ nuclear accumulation and the inhibition of the ARID1A-containing SWI/SNF complex (Chang et al., 2018). In other words, the ARID1A-containing SWI/SNF complex works as a mechano-regulated inhibitor of YAP/TAZ. In multiple experimental organoids, YAP/TAZ activity modulates stem cells' stemness and cell fates, which are eventually dictated by the spatio-temporal balance between material stiffness and degradability (Brusatin et al., 2018). Specifically, the stiffness-sensing YAP/TAZ is gradually inactivated due to the “contact inhibition” of cell proliferation; while this happens, the stem cells lose their stemness and choose a specific direction of differentiation. At this very moment, a suitable speed of biomaterial degradation could supply extra inner spaces for cellular proliferation thus preserving cellular stemness for organoid generation. These novel discoveries have highlighted how to spatio-temporally control stem cells differentiation through mechanically related YAP/TAZ activity and biomaterials degradation for best dermal matrixes.

As regards wound healing, experimental work uncovered an interesting “mechanical memory” effect: epithelial cells primed on stiff matrixes exhibited higher capabilities of migration and adhesion even after transfer onto a softer secondary matrixes (Nasrollahi et al., 2017). These interesting results showed that migrating cells can “remember” information from past physical environments and that these “mechanical memories” may be exploited to influence cell fates. As stem cells in chronic wounds show a slow self-renewal and remain uncommitted to differentiation, the tailored control of stem cells is believed to help wound healing.

Moreover, mechanical properties can influence scar formation. Reportedly, aberrant mechanotransduction is regarded as a driver of fibrosis (Brusatin et al., 2018). For chronic wounds, the mechanical signals might either affect extracellular matrixes remodeling through the “stiffness-sensing” capability of fibroblasts (Zhou et al., 2016) or impact the delivery of bioactive agents during the fibroblasts-to-myofibroblasts transdifferentiation, a key pathological process underlying the development of hypertrophic scars (Jiang et al., 2018).

Last but not least, the mechanical signals were also reported to regulate immune responses by influencing the behavior of human monocyte-derived macrophages (Adlerz et al., 2016), which is related to bacterial resistance in chronic wounds.

### Elastin-Based Dermal Matrixes

Due to the huge impact exerted by mechanical signals on the regulation of stem cell differentiation and of mechanotransduction in the course of wound healing, elastin-based dermal matrixes have recently emerged as means to provide elastic recoil and resilience to the wounded dermis and to prevent pathological scar retractions.

Generally, dermal elastic fibers and collagen fibers are responsible for Young's (elastic) modulus and the tensile strength of human dermis, respectively (Wang et al., 2015). In healthy skin, elastin monomers derive from the tropoelastin precursor and are then further crosslinked into polymeric networks. The latter enable healthy skin to recover its original shape once stretched to a great extent. In wounded skin, as compared to normal, a smaller amount of elastin monomers is produced, while most of the dermal fibers are deposited in an aberrant manner, resulting in a disorderly fibrous and poorly elastic network. Although elastin accounts for only about 2–4% of the human skin dry weight (Rodriguez-Cabello et al., 2018), its significant functional role is attracting a growing attention aimed at improving dermis mechanical properties and at regulating cellular activities during wound healing.

Currently, dermal matrixes trying to mimic the natural elastic properties of human skin are based on either natural tropoelastin or synthetic elastin-like recombinamers (ELRs) (Rodriguez-Cabello et al., 2018).

#### Tropoelastin-Based Dermal Matrixes

Tropoelastin, which naturally exists in all vertebrates except Cyclostomes, is a 60–72 kDa protein made of 750–800 amino acid residues constituting the dominant building block of the elastic fibers that imbue tissues with elasticity and resilience. Tropoelastin is made up of alternating hydrophilic and hydrophobic domains. The latter show elastic properties while the intercalated hydrophilic lysine-rich domains act as crosslinkers. As recently reported, the established atomic structure of human tropoelastin is an extended molecular body flanked by two protruding legs (Wang et al., 2015). The lysine residues of the hydrophilic domains show a substantial variation in their locations, which might contribute to their greater accessibility and cross-linking capacities (Tarakanova et al., 2019).

The versatile and pliable potentialities of tropoelastin have attracted interest from several biomedical fields. Tropoelastin and silk fibroin were blended to produce electrospun yarns, in which the elasticity of the former is combined with the mechanical strength of the latter. The results of the subcutaneous implantation of such yarns in mice proved their good tolerance and persistence for over 8 weeks, supporting their potential application to tissue engineering (Aghaei-Ghareh-Bolagh et al., 2019). In another study, the engineered tropoelastin-polydopamine-coated tendon scaffolds promoted the tenogenic commitment of human adipose tissue-derived stem cells which remarkably synthesized and deposited elastin in the generated elastin-rich matrixes *in vitro* (Almeida et al., 2019). Yeo et al. (2019) immobilized tropoelastin on plasma-coated polyurethane films, which significantly promoted the adhesion and proliferation of multipotent adult progenitor cells.

The first *in vivo* study focusing on the therapeutic effects of tropoelastin on full-thickness dermal wounds was recently reported by Mithieux et al. (2018). The implanted pure tropoelastin exhibited superior cell recruiting properties and significantly promoted angiogenesis, resulting in an enhanced healing of full-thickness pig skin wounds. The pure tropoelastin used for the experiments was dissolved, dried, and heated in a stepwise procedure with no addition of other chemicals. Although the regeneration included vessels, rete ridges, and a keratinizing stratified epithelium, no results were reported about the recovery of any degree of elasticity on the part of the tropoelastin-treated wounds.

Although the above discoveries highlighted the potential therapeutic values of tropoelastin as regards wound healing, we still lack *in vivo* evidences in terms of a restored elasticity of regenerated wound tissues during lengthy follow-up observations.

#### ELRs-Based Dermal Matrixes

ELRs are genetically engineered polypeptides having the repeated elastin sequence valine-proline-glycine-X-glycine (VPGXG), where X can be any amino acid excepting proline. The structures made of engineered ELRs are tunable and offer versatile elastic-tailored applications. Gonzalez de Torre et al. (2018) described the “clickable” properties of ELRs and used electrospinning to prepare bioactive fibers from clickable ELRs with no crosslinking agent added. In another study, Changi et al. (2018) developed thermo-sensitive ELRs which contained bioactive molecules and showed good biocompatibility and limited immunogenicity in BALB/c and C57BL/6 mouse models. Moreover, some functional sequences, such as growth factors, can be attached via chemical reactions to ELRs molecule or can be directly inserted into main sequences of ELRs through recombinant techniques (Flora et al., 2019).

To sum up, mechanical signals can regulate stem cells, scar formation, or immune responses during wound healing. Current studies have shown that both tropoelastin and ELRs exhibit a potential for chronic wound care. However, limited research has been hitherto focused on the recovered elasticity of the regenerated skin. Further experiments need to be carried out to confirm the long-term therapeutic effects of elastin- and ELRs-based dermal matrixes.

## Structural Properties

### Structure-Related Regulation During Wound Healing

Structural properties of dermal matrixes include pore sizes, porosity, surface topology, organization of inner frames, etc. Among structural properties, pore sizes (Wang et al., 2016) and porosity (i.e. the ratio between the hollow space inside a scaffold and its overall volume; Xu et al., 2016) have been extensively studied. Dermal matrixes presently available in clinical settings have suitable pore sizes and high degrees of inner connections to allow cell migration as well as nutrients exchange. The Wnt/β-catenin signaling pathway was recently reported to regulate pore-size-related cell proliferation (Xu et al., 2018). The current consensus is that ideal pore sizes or porosity have yet to be ascertained for the different kinds of matrixes and seeded cells (Xu et al., 2015).

Interestingly, surface topology was found to regulate stem cells mainly via roughness and texture (Xing et al., 2019). Firstly, a rough surface can attract stem cell aggregation. In human skin, stem cells usually gather and undergo self-renewal in “niches,” i.e., the interfollicular epidermis, the basal layers, and the hair follicle bulges (Alonso and Fuchs, 2006). Likewise, stem cells tend to aggregate on rough surfaces endowed with topologically “artificial niches,” such as holes, canyons, grooves, or craters (Cooper et al., 2012). Secondly, surface textures with different shapes further direct stem cell fates. Kilian et al. (2010) patterned the mesenchymal stem cell individually on a substrate and each cell was patterned with a certain shape, i.e. rectangles or pentagonal symmetries (five-pointed star). The mesenchymal stem cells were found to display different adipogenesis and osteogenesis profiles once seeded onto different shapes. Furthermore, the mesenchymal stem cells were then patterned individually onto different rectangles with increasing aspect ratios as well as onto dissimilar pentagonal symmetries with varied subcellular curvatures. Their results showed that changing the cell shapes correspondingly changed the cell lineages. In other words, altering the shapes of stem cells can influence the cell lineages they generate. Therefore, manipulating the roughness and texture of surface topology could enable us to tailor the fate of the individual cell.

Recently, surface micropatterning methods (i.e., nanotechnology, 3D bio-printing, laser photolithography, microcontact transfer method, electron beam etching, etc.) can manipulate the microgeographic structures on matrix surfaces (Bui et al., 2018). Therefore, surface micropatterning methods hold potential to regulate the stem cell fates during wound healing via tailoring the surface topology. In a recent study, a crossed groove/column micropattern was constructed on the surface of bacterial cellulose matrix using low-energy CO_2_ laser photolithography. Animal experiments indicated that this micropatterned shape guided a “basket-woven” organization of collagen distribution that may reduce scar formation (Hu et al., 2019).

**Figure 2:**
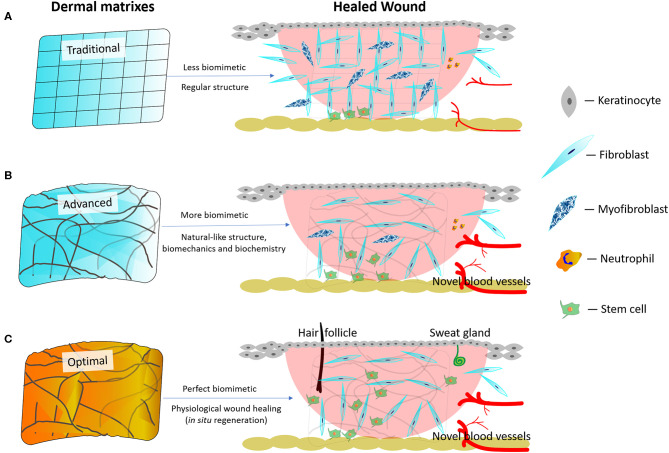
Schematic diagram of traditional, advanced, and best dermal matrixes and their respective therapeutic outcomes. **(A)** Traditional dermal matrixes create less biomimetic environments leading to a more abundant scar formation and fewer regenerated blood vessels. **(B)** Advanced dermal matrixes mimic the natural skin environment better than traditional dermal matrixes, thereby leading to a less abundant scar formation and a more intense regeneration of blood vessels. However, the regeneration of cutaneous appendages (i.e., hair follicles, sweat, and sebaceous glands) remains difficult to achieve. **(C)** Optimal dermal matrixes enable wounds to reach a complete tissue regeneration (theoretically physiological wound healing) *in situ*.

Anyhow, natural skin environment acts as the blueprint for engineered dermal matrixes. Maximally mimicking the natural skin environment holds potential to achieve a scarless regeneration *in situ* (MacEwan et al., 2017). Traditional dermal matrixes create less biomimetic environments that do not help commit stem cells to differentiation. Consequently, higher numbers of fibroblasts transdifferentiate into myofibroblasts, which possibly leads to hypertrophic scars (Figure 2A; Li Y. et al., 2019). Nowadays, advanced dermal matrixes endowed with more skin-like biomimetic architectures and mechanical properties as well as with the necessary biochemical signals are believed to commit stem cells to differentiation (MacEwan et al., 2017), which further reduces scar formation while promoting angiogenesis (Figure 2B). However, the regeneration of cutaneous appendages (i.e. hair follicles or sweat glands) is not yet satisfactory. Theoretically, optimal dermal matrixes should enable wounds to achieve tissue regeneration (i.e. physiological wound healing) *in situ*, which means that the healed wound has the same morpho-functional features as the natural skin, is devoid of scar tissue, and has concurrently regenerated the cutaneous appendages (i.e. hair follicles or sweat glands; Figure 2C). However, it is still beyond our sight what the best dermal matrixes might be and how they would regulate stem cells differentiation.

Further understanding of natural dermal structures might inspire the organization of the inner structures (e.g. random, aligned, gradient, porous, or filamentous) of dermal matrixes. Reportedly, collagen/elastin-based three-dimensional (3D) histological images indicated that the human dermis amounts to a “sandwich” structure with gradient changes in gradual terms of either interstitial spaces or architecture at different dermal depths (Wang et al., 2015). However, no solid conclusion has been hitherto reached whether dermal aligned or gradient structures are significantly better than random or homogeneous structures. The best patterns of the inner structures of dermal matrixes are yet to be assessed for the different cell types and chemical components.

### Concerns Regarding Mechanical and Structural Regulation of Cells

Although mechanical and structural properties of dermal matrixes have shown crucial effects on cell behaviors, we cannot yet reach a solid conclusion concerning one or another specific parameter and its corresponding therapeutic effects. Tunable mechanical and structural properties exert a previously undocumented influence on either the fate of each cell type or the whole wound healing process.

First, it is difficult to decouple the interplay between structural and mechanical properties. When we tune one physical property and study its therapeutic effects, inevitably other properties concurrently change. For example, when we tried to fabricate a series of scaffolds with varied stiffness gradients, the other intrinsic properties (e.g., pore size, porosity, organization of inner structures, and elastic modulus) changed correspondingly. One study reported that mixtures with different proportions of collagen and hydroxyapatite could be coated on decellularized cancellous bone to vary its stiffness with no statistically significant after-coating changes in the scaffold architecture. Notwithstanding this, the chemical composition and related cell binding sites underwent concomitant changes (Chen et al., 2015). In another study, the cryoprotectant dimethyl-sulfoxide was used to control pore size by regulating ice crystal sizes in 3D freeze-dried porous scaffolds, while the stiffness was regulated by adjusting the degree of cross-linking (Jiang et al., 2019). Although using the above methods achieved an independent control of pore size and stiffness, the decoupling of structural and mechanical properties affecting cellular activities was far from being satisfactory due to variations in chemical inhomogeneity among scaffolds.

Second, it is difficult to observe cellular responses to a single tunable physical property due to the complex and changeable spatio-temporal microenvironments, because so many kinds of cells are involved in wound healing and each cell type is regulated by a specific set of multiple factors. For example, porosity and pore size are two correlated physical properties. On the one hand, smaller pores favor cell adhesion and immigration because of their higher surface area and ligand density. On the other hand, smaller pores are easily subject to clogging when cells grow on their inside, the upshot being a decreased porosity which further reduces permeability to oxygen and nutrients. It seems that we cannot find the “best choice” for a single specific parameter due to the great complexity of cellular microenvironments.

Third, cellular behaviors distinctly differ going from individual cell level to tissue/organ level. Micropatterning technologies (Brusatin et al., 2018) have been intensively used to study individual cell's behaviors with respect to various physical properties in two dimensions (2D). By this way the first evidences and groundbreaking discoveries were mostly gained about mechanical-related cellular responses. However, from what is known about 2D cultures, it is difficult to extrapolate the cellular behaviors in complex 3D environments. Moreover, precisely tuning physical properties of 3D matrixes faces insurmountable difficulties due to the concomitant changes in the other parameters, just as we mentioned above.

Fourth, the confusion about bulk stiffness and local stiffness also causes concerns. Bulk stiffness is usually measured via tensile/compression tests and refers to the scaffolds overall macroscopic features. However, local stiffness is usually measured via atomic force microscopy and is believed to be the only biophysical signal scaffolds-attached cells can sense and respond to. A study using atomic force microscopy indicated that the local stiffness of different sites within acellular fibrotic lungs was very inhomogeneous (Melo et al., 2014). Therefore, when attached cells are migrating through a scaffold, they will experience significantly heterogeneous degrees of local stiffness from their own perspective. There are worrying trends that many studies of cellular mechano-responsiveness only focus on bulk stiffness, yet unintentionally neglect local stiffness. Concerning dermal matrixes, it is still unclear how local stiffness and bulk stiffness antagonistically regulate stem cell fates during wound healing.

Hopefully, many scientists have started taking heed of this problem. In order to mimic the dynamic microenvironments, a 4D programmable culture system with self-morphing capabilities was developed to regulate the controlled differentiation of neural stem cells (Miao et al., 2020). In addition, mathematical methods also help to solve these problems. Recently, a Bayesian linear regression mathematical model was used to predict the changes of topography-induced gene expression (Cutiongco et al., 2020). In another case, a mathematical model was used to assess the correlation between local and bulk stiffness, i.e., the bulk/local stiffness ratio. The results showed that the local stiffness detected by atomic force microscopy fell within the value ranges predicted via the mathematical model (Jiang et al., 2019).

## Size-related Properties

Nanotechnology has become known as an exciting wound treatment tool. Multiple kinds of macroscopic nanobiomaterials (e.g., electrospun nanofibers, nanosheets, nanoemulsions, carbon nanotubes-based, or graphene-based nanocomposites) and nano-sized biomaterials (e.g., NPs, ions, molecules, nucleic acids, functional peptides, proteins, oligosaccharides, or polysaccharides) have exhibited great potential capabilities of modulating vascularization, bacterial resistance, and inflammation during wound healing (Chakrabarti et al., 2019).

In this regard, size-related physical properties targeting chronic wound care are anti-infection outcomes contributed by NPs. Common NPs in use are metals (e.g., Ag, TiO_2_, ZnO, MgF_2_, CeO_2_), lipid-based vesicles (e.g., liposome, exosomes), and polymers. Compared with materials of regular sizes, NPs quite differ in regard to mechanical strengths, melting points, surface areas, optical, and magnetic properties (Das and Baker, 2016).

### Metal NPs

The silver NPs (AgNPs) are the most widely used metal NPs in both laboratory and clinical applications. AgNPs ranging from 1 to 100 nm in size or silver nanoclusters with an ultrasmall size (<2 nm) have shown good antimicrobial properties. Solid evidence has been provided that AgNPs can prevent and/or fight microorganism infections and significantly enhance the healing of chronic wounds (Sandri et al., 2019).

Bacterial biofilms are associated with the resistance to an extensive range of antibiotics, contributing to chronic wounds formation. Recent studies have shown that AgNPs exert promising therapeutic effects against the biofilm-forming MRSA (Zhang et al., 2018). Another study also reported the inhibiting ability of AgNPs loaded with thymol and chitosan on the biofilm formation by MRSA with a 10.08 ± 0.06 mm zone of inhibition (ZOI) and a minimum inhibitory concentration of 100 μg mL^−1^ (Manukumar et al., 2017). The AgNPs killing efficacy on biofilms of *Vibrio* species, another group of clinically multi-drug resistant bacteria, was also verified (Satish et al., 2017). Moreover, clinical data further confirmed the effectiveness of AgNPs against biofilm-forming bacteria. Thomas et al. (2014) isolated from clinical samples a series of multidrug-resistant biofilm-forming coagulase-negative staphylococci, e.g. *S. epidermidis* strains, *S. aureus, Salmonella typhi*, and *Salmonella paratyphi*. Surprisingly, AgNPs exerted their antibacterial activity against all the tested strains. A randomized and double-blind pilot clinical trial study also revealed that Nano Silver Fluoride particles can inhibit the formation of *Streptococcus* mutants' biofilms (Freire et al., 2017).

The likely underlying mechanisms of AgNPs' antibacterial properties lie in structurally damaging the cell membranes and in deeply altering the intracellular metabolic activities of the bacteria (Eckhardt et al., 2013). AgNPs also inhibit the activities of bacterial respiratory enzymes (Franci et al., 2015). In addition, other evidences demonstrated that AgNPs control inflammatory processes and modulate cytokines' activities (Rigo et al., 2013), which might be additional mechanisms benefiting chronic wound healing.

### Lipid NPs

Liposomes, also known as phospholipid vesicles, are the most widely used lipid NPs for wound dressings (Nasab et al., 2019). In contact with an aqueous solution liposomes can be automatically assembled into enclosed phospholipid bilayers containing a watery core surrounded by a hydrophobic membrane (Ahmed et al., 2019). Reportedly, vancomycin-loaded nanoliposomes coupled with an anti-staphylococcal protein (lysostaphin) can serve as potential antimicrobial formulations for wound infections caused by MRSA, which is resistant to several conventional antibiotics (Hajiahmadi et al., 2019).

The lipid-based NPs versatile capabilities due to their nanoscales, biocompatibility, and high permeability might change our classical views on drugs pharmacokinetics. For example, egg lecithin and soy lecithin liposomes showed superior antioxidant activity *in vitro* and significantly accelerated wound-healing *in vivo* (Nasab et al., 2019). Reportedly, farnesyl-encapsulated liposomes promoted wound healing in a rat model with third-degree burns (Wu et al., 2019). More interestingly, liposomes encapsulating propolis, a natural bee product, showed both antimicrobial and antioxidant activities (Aytekin et al., 2019), which indicated the multiplicity of potential applications of liposomes in pharmacotherapy.

Besides discovering the potential therapeutic effects of new drugs, lipid-based NPs might also enable us to amplify the applications of traditional drugs regarding chronic wound care. Insulin administered by injection is a drug used to regulate blood sugar levels in Internal Medicine. Recently, the promising therapeutic effects of insulin's external administration on chronic wounds were noted, probably because insulin regulates nutrients' metabolism further helping cellular activities during wound healing. However, insulin's external administration is a great technical challenge due to its limited transdermal absorption and its rapid degradation in the wound's bed. To solve this problem, Dawoud et al. (2019) formulated insulin-loaded chitosan NPs liposomes which successfully prolonged the release of insulin.

Moreover, the use of liposomes for intracellular drug delivery is a promising approach to reduce scarring, promote vascularization of ischemic wounds or regulate inflammation in cases of diabetic ulcers and other types of chronic wounds (Choi et al., 2017). Reportedly, glucocorticoid-loaded liposomes did induce a pro-resolution phenotype in human primary macrophages, which significantly promoted the healing of chronic wounds (Gauthier et al., 2018). Similarly, fibroblast growth factor-encapsulating liposomes advanced wound healing in rats (Xiang et al., 2011). Nunes et al. (2016) developed an usnic acid/liposomes-embedded gelatin-based membrane which is capable of transdermal absorption by skin layers and of controlled drug release. Another intracellular delivery use of liposomes aimed at upregulating growth factor co-receptors in diabetic wounds, which usually heal with difficulty to due to growth factor resistance (Das et al., 2014).

Other widely used lipid NPs are exosomes (Chen et al., 2019), solid lipid NPs, and more. Exosomes have diameter sizes ranging from 30 to 100 nm, and are usually released from cells when multivesicular bodies fuse with the plasma membrane (Zarrintaj et al., 2017). Reports indicated that exosomes derived from gene-modified microRNA-126-overexpressing synovium MSCs significantly promoted the proliferation of human dermal microvascular endothelial cells and of human dermal fibroblasts in a dose-dependent manner (Tao et al., 2017). Besides, solid lipid NPs (Eskiler et al., 2019) also exhibited superior capabilities of controlling drugs delivery for potential wound care applications. Moreover, lipid-based NPs partake in emerging applications in relation to several fields, such as cancer therapy, vaccines, dermatological treatments, ocular delivery (Li N. et al., 2019), and post-surgery pain control (Cohen et al., 2019).

### Polymeric NPs

Polymeric NPs are usually used to encapsulate drugs, nucleic acids, proteins, macromolecules, and growth factors in order to extend their half-life and improve their bioavailability by physically isolating them from the wound's bed environment, in which multiple kinds of proteases are present. Moreover, the sustained release of drugs at therapeutic concentrations not only can reduce the frequency of drug deliveries but also achieves optimized pharmacokinetics profiles (Kim et al., 2018). Therefore, wound dressings with polymeric NPs have drawn increasing attention due to their intracellular delivery capabilities.

Among them, poly (lactic-co-glycolic acid) (PLGA)-based NPs are widely used for controlled drug releasing due to their versatile degradation kinetics. PLGA biodegradation can also release lactate byproducts further advancing wound healing processes. An endogenous human host defense peptide, LL37, was encapsulated into PLGA NPs to prevent infection and accelerate wound healing (Chereddy et al., 2014). Karimi Dehkordi et al. (2019) formulated a nanocrystalline cellulose-hyaluronic acid composite embedded with granulocyte-macrophage-colony-stimulating-factor-loaded chitosan NPs to promote wound healing. In the last composite, nanocrystalline cellulose acts as a strengthening agent boosting the mechanical properties of hyaluronic acid.

As biological macromolecular compounds, polymeric NPs exhibit bioactivities that are easily affected by several physical properties, such as sizes, components, surface charges, shapes, etc. Hasan et al. (2019) developed positively- and negatively-charged, respectively, PLGA NPs containing the antibiotic Clindamycin. Although both kinds of nanoparticles did no detectable harm to healthy fibroblasts, the positively charged NPs elicited significantly better therapeutic outcomes of wound healing in a MRSA-infected mouse model. The different effectiveness in antibacterial activity exhibited by the positively charged *vs*. the negatively charged polymeric NPs was probably due to their dissimilar capability of adhering to bacteria. To systematically compare the effects of NPs surface charges and shapes on wound healing, Mahmoud et al. (2019) synthesized a series of polymeric hydrogels loaded with gold NPs of differing shapes (rods and spheres) and introduced various surface modifications (neutral, cationic, and anionic charged polymers). Both the inherent parameters (e.g., colloidal stability and release behavior) and therapeutic outcomes (e.g., wound healing, skin re-epithelialization, collagen deposition, inflammation level, and antibacterial activity) were assessed in an animal wound model. Their results showed that hydrogels of gold nanorods constitute a promising nano-platform for wound healing.

Like lipid-based NPs, polymeric NPs might also affect drugs' pharmacokinetics in wound environments, thus enabling us to explore potential off-label uses of current drugs. Jia et al. (2018) developed nanofibrous PLGA incorporating andrographolide-loaded mesoporous silica NPs. Their results showed that the sustained releasing of andrographolide, which is extracted from a Chinese herb, surprisingly reduced inflammation intensity while promoting epidermal cells adhesion. Another kind of Chinese herb, *Aloe vera*, was reported to enhance wound healing when encapsulated inside PLGA NPs to be used in wound dressings (Garcia-Orue et al., 2019). A further recent example comes from the interesting works of Farghaly Aly et al. (2019) who showed that hydrogels containing polymeric NPs loaded with Simvastatin, a cardiovascular drug commonly used to treat serum hyperlipidemia, can benefit wound healing processes. Once encapsulated in chitosan NPs, Phenytoin, an antiepileptic drug, also exhibits a potential capability to accelerate wound healing, a suggestion inspired by recently reported clinical cases of gingival hyperplasia after Phenytoin's oral administration (Cardoso et al., 2019).

### Biosafety Concerns of NPs

The biosafety and cytotoxicity of AgNPs have already aroused many concerns. AgNPs seem to be cytotoxic for many species including human beings (Shavandi et al., 2011; Pratsinis et al., 2013). Newly published research showed the toxicity of AgNPs on marine microalgae (Hazeem et al., 2019) and the yeast *Saccharomyces cerevisiae* BY4741 (Kasemets et al., 2019). As regards human beings, prolonged exposures to AgNPs can cause argyria (Richter et al., 2015), whose symptoms include blue gray skin color changes and multiple alterations of bodily functions such as gastrointestinal disorders, spasms, and even death (Rice, 2009). Importantly, AgNPs have produced genotoxicity in the testicles of Sprague Dawley rats (Elsharkawy et al., 2019) and in the embryos of Zebrafish (Chakraborty et al., 2016). As regards human beings, AgNPs have showed genotoxicity in human liver HepG2 and colon Caco2 cells (Sahu et al., 2016). These results revealed a possible reproductive genotoxicity of AgNPs on human offspring especially when AgNPs are used at high doses and for lengthy periods in patients with large area burns or chronic wounds. Moreover, AgNPs can possibly induce neurotoxicity by crossing the brain blood barrier and penetrating the central nervous system of human beings (Khan et al., 2019).

To test the toxicity when AgNPs are applied on wounds, Pang et al. (2020) applied AgNPs onto the wounds of Zebrafish after the amputation of fins. AgNPs were found to impair epithelialization and blastema formation especially during the first few days, showing that the cytotoxicity of AgNPs is time-dependent and is more obvious at the initial stages of wound healing. Konop et al. (2019) used micellar electro kinetic chromatography to detect the releasing profile of AgNPs from wound dressings. AgNPs at concentration higher than 10 ppm exerted significant (*p* < 0.05) toxicity on the fibroblasts isolated from diabetic mice vs. a murine fibroblasts cell line and a human fibroblasts cell line. Our team also found that an exposure to high concentrations of AgNPs significantly inhibited the proliferation of mice fibroblasts (Liu et al., 2018b). Specially, we noticed that the antibacterial efficiency stopped growing and entered a plateau stage as the AgNPs doses increased, which indicated that an optimized dose range does exist for AgNPs (Liu et al., 2017).

Also, attentions have already been paid to the biosafety and toxicity of other metal NPs, such as TiO_2_, ZnO, magnesium fluoride (MgF_2_), cerium oxide (CeO_2_), copper, iron oxide, gold, etc. Wang et al. (2018) compared the toxicity of Ag, TiO_2_, and ZnO NPs to human smooth-muscle cells. Their results showed that all the three kinds of metal NPs could induce inflammatory responses. More importantly, ZnO NPs significantly increased intracellular ROS showing a stronger cellular cytotoxicity than that of Ag and TiO_2_ NPs. In another case, Filipova et al. (2018) reported a “three-in-one” screening assay to test the toxicity of three kinds of NPs for human umbilical vein endothelial cells. The three NPs are silica NPs (7–14 nm), superparamagnetic iron oxide NPs (8 nm), and carboxylated multiwall carbon nanotubes (60 nm), all of which were tested at the same concentration of 100 μg/ml. Surprisingly, all the NPs types tested exhibited a gradual toxic effect which decreased cell viability.

### Solutions to Reduce Cytotoxicity of AgNPs in Wound Care

Reportedly, a general mechanism for AgNPs-mediated intracellular toxicity is that AgNPs can enter human cells either by endosomal uptake or by diffusion (Frohlich and Frohlich, 2016). The cytotoxicity of AgNPs is a size-, dose-, and time-dependent, which means that it is closely related to nanoparticle size, shape, surface charge, oxidation state, agglomeration condition, administration route, and dosage (Liao et al., 2019). Correspondingly, potential solutions to reducing cytotoxicity of AgNPs are focused on the tunable size-, dose-, or time-dependent features of AgNPs.

First, increasing the size of AgNPs can reduce their cytotoxicity, but the antibacterial efficacy is concurrently reduced. Reportedly, AgNPs with sizes below 10 nm exhibited both a higher antibacterial efficiency and cytotoxicity (Gahlawat et al., 2016). Zille et al. (2015) tested the antibacterial efficacy of AgNPs with 10, 20, 40, 60, and 100 nm particle sizes. The antibacterial inhibition values against *S. aureus* were 19% for the 100 nm-AgNPs and 95% for the 10 nm-AgNPs, showing that antibacterial effectiveness increases with decreasing nanoparticle size. On the other hand, AgNPs of sizes below 10 nm showed to be more cytotoxic than those of other sizes (Recordati et al., 2016). AgNPs of sizes below 3 nm can be deposited within multiple organs of male mice, such as liver, spleen, kidney, heart, lungs, testicles, stomach, and intestine (Yang et al., 2017). Therefore, a balance needs to be achieved between antimicrobial efficiency and biosafety due to the size-dependent cytotoxicity of AgNPs.

Second, combining AgNPs with other antibacterial strategies can reduce the administered doses to lessen the dose-dependent cytotoxicity. For example, antimicrobial peptides (AMPs), which are integral compounds secreted by natural organisms and act as natural immune defenses (Kokel and Torok, 2018), can possibly reinforce AgNPs in terms of killing antibiotic-resistant bacteria. In a recent study, AMPs were included at the peripheral hydrophilic region of polymersomes, while AgNPs were included inside the hydrophobic corona. *In vitro* tests indicated that the AMP/AgNPs polymersomes exhibited a satisfactory bacteriostatic activity as well as a low cytotoxicity toward human dermal fibroblasts (Bassous and Webster, 2019). In other cases, AgNPs-coated zwitterionic hydrogels, which confer superhydrophilic properties to resist bacterial attachment, were reported to promote wound healing (GhavamiNejad et al., 2016). Our team recently used N,N-dimethylformamide-treated poly(vinylidene fluoride), in which the Ag^+^ ions were reduced to elemental silver. The impregnated AgNPs were then generated *in situ* and the surface hydrophobicity was significantly increased. Then, we tried different concentrations of ingredient materials and eventually reduced the cytotoxicity and achieved optimized anti-bacterial capacities against *A. baumannii* and *E. coli* (Menglong Liu et al., 2018).

Third, novel drug delivery system can release AgNPs in a spatio-temporally controlled or stimuli-responsive profile to reduce dosing frequency and amounts. As regards chronic wounds, the stimuli which trigger the release of AgNPs can be pH levels, lactic acid, glucose, proteases, and matrix metalloproteinases, etc. Among all the peculiar stimuli, pH changes play a vital role by revealing pathophysiological alterations occurring during the transformation of acute wounds into chronic wounds due to infection, ischemia, or inflammation. Recently, our team reported the pH-responsive releasing of AgNPs reinforced via a chemo-photothermal therapy targeting chronic wounds with bacterial infections. The infectious wound environment (pH ~ 6.3) can trigger the release of AgNPs, which contributes to lower their cytotoxicity (Liu et al., 2018a).

## Overview

Obviously, stem cell fates are spatio-temporally modulated by concurrent signals from both physical and chemical stimuli. Surface micropatterning methods, especially the nanotechnology, 3D bio-printing, and laser photolithography enable us to precisely tailor the surface topology and biomechanical features to study the behaviors of individual cells. These discoveries highlighted that in some cases physical properties can be the predominating and independent factors that direct stem cell fates (Kilian et al., 2010).

Earlier strategies for the regeneration of cutaneous appendages (i.e., sweat glands and hair follicles) were focused on chemical approaches, which entailed insurmountable difficulties. The physical property-based regulation of stem cells has been tracking down potential solutions. For example, 3D bio-printed dermal matrixes served as “artificial niches” to direct epidermal progenitors (Liu et al., 2016) and MSCs (Yao et al., 2020) into sweat gland differentiation.

Traditional fabrication techniques do not tailor spatial structures or control modeling for the regenerations of human cutaneous appendages. Recently, a microfluidic device, which can manipulate fluids at the submillimeter scale (Sackmann et al., 2014), was ingeniously designed to recapitulate the development of human epiblast and amniotic ectoderm using human pluripotent stem cells (Zheng et al., 2019). This study showed that the guides of biomimetic spatial structures and physical cues play key roles during the regeneration of organoids with complex microstructures and microfluidics technology holds potential for the regenerations of human cutaneous appendages.

## Author Contributions

YW provided the main concept for this review and wrote the manuscript. UA and JW prepared the figures and improved the language of the manuscript.

## Conflict of Interest

The authors declare that the research was conducted in the absence of any commercial or financial relationships that could be construed as a potential conflict of interest.

## Abbreviations

AgNPs: silver nanoparticles
ARID1A: AT-rich interactive-domain 1A
ELRs: elastin-like recombinamers
MRSA: methicillin-resistant *Staphylococcus aureus*
MSCs: mesenchymal stem cells
NPs: nanoparticles
PLGA: poly(lactic-co-glycolic acid)
ROS: reactive oxygen species
SWI/SNF: switch/sucrose non-fermenting
TAZ: transcriptional co-activator with PDZ-binding motif
YAP: yes-associated protein.
